# Trends in breast cancer mortality attributable to metabolic risks in Chinese women from 1990 to 2019: an age-period-cohort analysis

**DOI:** 10.3389/fonc.2024.1369027

**Published:** 2024-04-16

**Authors:** Ting Zhang, Simeng Sun, Ting Xia, Qiaoyu Huang, Yali Fu, Weiwei Wang, Huafeng Yang, Xin Hong, Nan Zhou, Hao Yu

**Affiliations:** ^1^ Department of Science and Education, Nanjing Municipal Center for Disease Control and Prevention, Nanjing, Jiangsu, China; ^2^ Monash Addiction Research Centre, Monash University, Frankston, VC, Australia; ^3^ Department of Epidemiological Research, Jiangsu Health Development Research Center, Nanjing, Jiangsu, China; ^4^ Department of Noncommunicable Chronic Disease Control and Prevention, Nanjing Municipal Center for Disease Control and Prevention, Nanjing, Jiangsu, China; ^5^ Division of Medical Affairs, Nanjing Municipal Health Commission, Nanjing, Jiangsu, China; ^6^ Department of Noncommunicable Chronic Disease Control and Prevention, Jiangsu Provincial Center for Disease Control and Prevention, Nanjing, Jiangsu, China

**Keywords:** metabolic risks, high fasting plasma glucose, high body-mass index, breast cancer, age-period-cohort analysis

## Abstract

**Objective:**

Metabolic risks (MRs) are the primary determinants of breast cancer (BC) mortality among women. This study aimed to examine the changing trends in BC mortality associated with MRs and explore how they related to age, time period, and birth cohorts in Chinese women aged 25 and above.

**Methods:**

Data were sourced from the Global Burden of Disease Study 2019 (GBD2019). The BC mortality trajectories and patterns attributable to MRs were assessed using Joinpoint regression. The age-period-cohort (APC) model was employed to evaluate cohort and time period effects.

**Results:**

The age-standardized mortality rate (ASMR) of BC mortality linked to MRs displayed an escalating trend from 1990 to 2019, demonstrating an average annual percentage change (AAPC) of 1.79% (95% CI: 1.69~1.87). AAPCs attributable to high fasting plasma glucose (HFPG) and high body mass index (HBMI) were 0.41% (95% CI: 0.32~0.53) and 2.75% (95% CI: 2.68~2.82), respectively. APC analysis revealed that BC mortality due to HBMI in women aged 50 and above showed a rise with age and mortality associated with HFPG consistently demonstrated a positive correlation with age. The impact of HBMI on BC mortality significantly outweighed that of HFPG. The risk of BC mortality linked to HBMI has steadily increased since 2005, while HFPG demonstrated a trend of initial increase followed by a decrease in the period effect. Regarding the cohort effect, the relative risk of mortality was greater in the birth cohort of women after the 1960s of MRs on BC mortality, whereas those born after 1980 displayed a slight decline in the relative risk (RR) associated with BC mortality due to HBMI.

**Conclusion:**

This study suggests that middle-aged and elderly women should be considered as a priority population, and control of HBMI and HFPG should be used as a primary tool to control metabolic risk factors and effectively reduce BC mortality.

## 1 Introduction

Breast cancer (BC) is one of the most prevalent malignancies affecting women globally. According to the Global Cancer Statistics 2020 (GLOBOCAN 2020), 2.3 million new BC cases were reported in 2020, constituting 11.7% of all cancer incidences worldwide. Female BC incidence has surpassed lung cancer, becoming the foremost prevalent cancer globally for the first time (1, 2). BC ranks as the fifth leading cause of cancer-related deaths worldwide, with 110 out of 185 countries and territories experiencing the highest mortality from this disease, as reported by GLOBOCAN (3). Over the past three decades, China has witnessed a significant increase in the disease burden of female BC, with the incidence tripling (14.14/10,000 vs. 52.81/10,000) and the mortality nearly doubling (7.22/10,000 vs. 13.40/10,000) from 1990 to 2019. In 2020, China reported 420,000 new cases and 117,000 deaths attributed to female BC, ranking first in both incidence and mortality globally among female cancers (3, 4).

Risk factors associated with BC were well documented including physiological conditions, family history, metabolic risk, lifestyle factors, etc (5). In recent years, metabolic risks (MRs) have shown a growing influence on cancer-related deaths worldwide (6, 7) due to rapid economic and social development which has brought dramatic changes in the dietary structure and behavior of the population (8, 9). China is also facing a significant burden of diseases linked to MRs among G20 nations (10), particularly in individuals over 60 years old. The Global Burden of Disease (GBD) 2019 classifies MRs associated with BC into high fasting plasma glucose (HFPG) and high body-mass index (HBMI). Elevated MRs are reported to be closely correlated with heightened BC mortality risk. Over the last three decades, BC mortality attributed to MRs has surpassed behavioral risks, emerging as the leading risk factor for BC in Chinese women (6, 11, 12).

Despite substantial efforts have been dedicated to exploring changes in BC mortality over time and age-specific mortality (13, 14) and investigating various associated risk factors (15–18), there is a lack of comprehensive analysis on the specific impact of MRs on BC mortality in Chinese women in terms of time trends and cohort effects. In this study, we analyzed pertinent data from GBD 2019, employing an age-period-cohort (APC) model (19) to delineate the temporal trends of BC mortality attributed to MRs (HFPG and HBMI). The study provides a powerful window into BC mortality attributable to MRs and its temporal trends and cohort effects in Chinese women over the past three decades, and the findings may provide valuable reference points for MR management, BC prevention, and treatment strategies to some extent.

## 2 Materials and methods

### 2.1 Data sources

Data for this study were obtained from GBD 2019. The GBD 2019 database sources information from national disease surveillance systems, cause of death registration and reporting systems, and injury detection systems across 204 countries or regions, estimating the disease burden for 369 diseases, 87 risk factors, and injuries (20, 21). Specifically, we limited the data concerning MRs associated with BC in Chinese women aged ≥25 from 1990 to 2019. GBD Results Tool on the Global Health Data Exchange (GHDx) online platform was used as the data source for this study (https://vizhub.healthdata.org/gbd-results/, accessed on September 30, 2023). Specific data were derived from the GBD database using strategies of assigning “China” as the location, “Breast Cancer” as the cause, “Metabolic Risk” as the risk factor, and “Mortality” as the measure. Due to that all information had been de-identified and publicly available, ethical approval was exempted for our study.

According to GBD 2019, MRs have six main manifestations, including HFPG, high LDL cholesterol, high systolic blood pressure, HBMI, low bone mineral density, and kidney dysfunction (22). In GBD 2019, MRs linked to breast cancer in women include HFPG and HBMI. HFPG was defined as serum fasting glucose levels from 4.8 to 5.4 mmol/L, while HBMI was defined as a BMI from 20 to 25 kg/m² for adults aged 20 years and above (22).

The data were categorized into ten age groups by a 5-year band (25-29 years, 30-34 years,… >70 years), and periods were divided into six consecutive 5-year periods (1990-1994, 1995-1999,… 2015-2019). Groups under 25 years were excluded due to a small number of deaths. Data collation and summary were conducted using EXCEL 2016 and SPSS 23.0, while GraphPad Prism 9.5 software was used for infographics.

### 2.2 Statistical analyses

Joinpoint regression analysis was employed to evaluate the trends of BC-attributable mortality, calculating the annual percentage change and its corresponding 95% Confidence interval (CI) (23). The average annual percentage change (AAPC) was derived by computing the weighted mean of various annual percentage change values (24). The hypothesis test was implemented to determine whether the AAPC significantly deviated from zero. AAPC > 0 means an increasing trend, while AAPC< 0 means a decreasing trend during the segment (25). Joinpoint 5.0 software provided by the National Cancer Institute was utilized for this analysis.

The Age-Period-Cohort (APC) model was employed to assess population risk in specific years and the cumulative effects of health risks since birth. This model allowed the extraction of mortality data and analysis of the independent effects of age, time period, and birth cohort on the time trends in BC mortality attributable to MRs (HFPG and HBMI). The age effects were risks linked to outcomes of aging specific to individuals. The period effects were any outcomes linked with living during a particular period in all age groups, including control and prevention strategies, policies, and regulations. The cohort effects were any changes in outcomes between groups with the same birth years, presumably arising from differences in lifestyle and exposure degrees to risk factors (26). The general logarithmic linear form (27, 28) of the APC model is


(1) $$\rho =\alpha_{a}+\beta_{p}+\gamma_{c}$$

Where ρ is the expected incidence rate, and $\alpha_{a}$ , $\beta_{p}$ , and $\gamma_{c}$ indicate the effects of age, period, and cohort, respectively. It can be transformed into the form of age-period (Equation 2) and age-cohort (Equation 3):


(2) $$\rho_{\text{a}p}=\mu +(\alpha_{L}-\gamma_{L})(a-\bar{a})+(\pi_{L}+\gamma_{L})(p-\bar{p})+{\tilde{\alpha }}_{a}+{\tilde{\pi }}_{p}+{\tilde{\gamma }}_{c}$$

(3) $$\rho_{\text{a}c}=\mu +(\alpha_{L}+\pi_{L})(a-\bar{a})+(\pi_{L}+\gamma_{L})(c-\bar{c})+{\tilde{\alpha }}_{a}+{\tilde{\pi }}_{p}+{\tilde{\gamma }}_{c}$$

Where $\alpha_{L}-\gamma_{L}$ represents cross-sectional trend; $\alpha_{L}+\pi_{L}$ represents longitudinal age trend; $\pi_{L}+\gamma_{L}$ represents net drift; and ${\tilde{\alpha }}_{a}$ , ${\tilde{\pi }}_{p}$ and ${\tilde{\gamma }}_{c}$ represent age, period, and cohort deviation, respectively (27, 28).

The fitted APC model estimated many useful estimable functions. This study mainly focused on the following estimable functions: Net drift, which is the overall logarithmic linear trend by period and birth cohort, was used as an estimate of the overall AAPC for the outcome measure; conversely, the local drift was the logarithmic linear of each age group by period and birth cohort that reflected the AAPC of outcome measures in each age group. A drift of a minimal ±1% per year indicated a significant change in mortality (19). The longitudinal age curve was plotted to calculate the fitted age-specific rates adjusted for period deviations in a reference cohort, thus reflecting age effects. The period (or cohort) rate ratio (RR) was the ratio of age-specific rates value in each period (or cohort) relative to the reference period (or cohort), thus reflecting period (or cohort) effects (26, 29). This study used the APC model analysis tool developed by the Biostatistics Branch of the National Institutes of Health.

## 3 Results

### 3.1 Trends in BC deaths attributable to MRs

**Table 1**, **Figures S1, S2A-C** illustrate the trends in BC mortality and the joint regression output attributed to MRs (HFPG and HBMI) among Chinese women over the past three decades. The number of deaths and mortality attributed to MRs related to BC has shown a substantial increase. The most prominent rise was observed in the number of deaths (AAPC: 4.97%; 95% CI: 4.89-5.07) attributed to MRs, representing an approximately 2.4-fold increase in the crude mortality from the whole study period. The age-specific mortality rate (ASMR) increased from 0.93/10^5^ to 1.55/10^5^ with a consistent increase from 1990 to 2019 (P<0.05). Further analysis of BC deaths attributed to HFPG and HBMI revealed consistently higher numbers and mortality related to HBMI compared to HFPG. The number of BC deaths attributed to HBMI increased by 5.5-fold in 2019 compared to 1990, with an AAPC of 6.06% (95% CI: 5.98~6.14) (**Table 1**). Between the period of 1998 to 2004, we witnessed the biggest growth with an APC of 4.57% (95% CI: 3.94~5.14). The AAPC of ASMR for BC attributed to HFPG and HBMI was 0.41% (95% CI: 0.32~0.53) and 2.75% (95% CI:2.68~2.82), respectively (**Figure S1**). BC attributed to HBMI largely reflected the overall MRs trend, while BC linked to HFPG showed a slower, fluctuating increase over the three decades (**Figures S2A-C**).

**Table 1:** Number of Deaths, Crude mortality and age-standardized mortality per 100,000 of BC attributable to MRs (HFPG and HBMI) in 1990 and 2019, and its temporal trends from 1990 to 2019.

	Index	1990	2019	AAPC (%)
MRs	Number of Deaths (n)	2142	17512	4.97*(4.89~5.07)
	Crude mortality (/10^5^, 95%CI)	0.70(0.22~1.40)	2.40(0.90~4.30)	4.27*(4.19~4.36)
	ASMR (/10^5^, 95%CI)	0.93(0.29~1.86)	1.55(0.58~2.79)	1.79*(1.69~1.87)
HFPG	Number of Deaths (n)	1931	5453	3.58*(3.50~3.67)
	Crude mortality (/10^5^, 95%CI)	0.34(0.06~0.79)	0.78(0.14~1.85)	2.89*(2.80~2.99)
	ASMR (/10^5^, 95%CI)	0.45(0.09~1.05)	0.52(0.10~1.23)	0.41*(0.32~0.53)
HBMI	Number of Deaths (n)	2191	12059	6.06*(5.98~6.14)
	Crude mortality (/10^5^, 95%CI)	0.38(0.07~0.93)	1.73(0.49~3.61)	5.34*(5.27~5.42)
	ASMR (/10^5^, 95%CI)	0.51(0.09~1.23)	1.11(0.31~2.33)	2.75* (2.68~2.82)

CI, confidential interval; AAPC, average annual percentage change; *Statistically significant (p< 0.05).

### 3.2 ASMR for BC attributable to MRs by age and period

**Figure 1:**
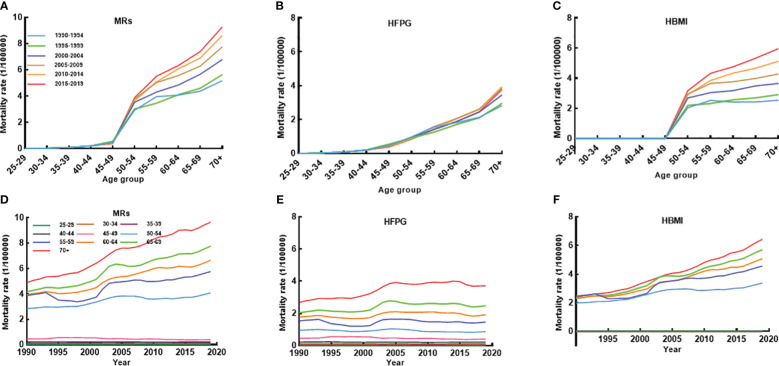
The age-specific mortality of BC attributable to MRs (HFPG and HBMI) between 1990 and 2019 and cohort-specific mortality of BC attributable to MRs by age groups between 1990 and 2019. **(A–C)** The survey years were arranged in five consecutive years (1990-1994, 1995-1999, 2000-2004, 2005-2009, 2010-2014, 2015-2019), and age-specific mortality was the average of the five consecutive years of mortality for that age group. **(D–F)** Trends in mortality between 1990 and 2019 by 10 different age groups.

**Figure 1**, **Tables S1, Figures S2D-F** further demonstrate BC mortality attributable to MRs (HFPG and HBMI) by age, period, and AAPC for age-specific ASMR from 1990 to 2019. BC mortality attributable to MRs, particularly HBMI, showed a clear escalation among women aged 50 years and older during this period (**Figures S2D–F**). The mortality showed a clear upward trend with increasing age. BC mortality linked to MRs and HBMI displayed higher rates after age 50 throughout the three decades (**Figures 1A–C**). Moreover, age-specific BC mortality attributed to HBMI paralleled the overall MRs trend, while BC mortality associated with HFPG had a strong positive relationship with age, with higher age-specific mortality during the same time period (**Figures 1D–F**). The AAPC for age-specific BC mortality attributable to MRs steadily rose from 1990 to 2019 from age 50. In 2019, BC mortality in women aged 70 years or older was approximately twice as high as in 1990 (AAPC: 2.31%; 95% CI: 2.25 to 2.38). The rise in mortality due to HBMI was more pronounced, with its AAPC for mortality in women over 50 surpassing the AAPC for MR-induced mortality in the same age group (**Tables S1**).

### 3.3 Trends of BC mortality in age groups

**Figure 2:**
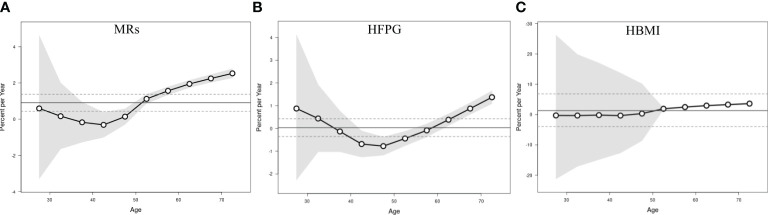
Local drift with net drift values of the mortality for BC attributable to MRs (HFPG and HBMI) in China from 1990 to 2019. **(A)** Corresponding to MRs; **(B)** Corresponding to HFPG; **(C)** Corresponding to HBMI. The dots and shaded areas denote percentage and their corresponding 95%CI.

**Figure 2** illustrates the net drift and local drift representing the overall AAPC for the entire study period and for each age group. The net drift of ASMR attributed to MRs among Chinese women aged 25 or older from 1990-2019 was estimated at 0.91% (95% CI: 0.44-1.38), indicating a statistically significant overall upward trend in BC mortality linked to MRs over the study duration. Regarding local drift, mortality attributable to MRs in the age group of 25-45 years displayed a declining trend with age, whereas in women aged above 45, it increased progressively. The local drift of mortality due to HBMI began to be greater than 0 after age 45 years, while the local drift linked to HFPG did not show an upward trend greater than 0 until after age 60 years. This indicates that in the past three decades, BC mortality attributable to MRs (HBMI and HFPG) has been significantly increasing in middle-aged and old-aged women, and the largest increases were in women over 70 years old, with AAPCs of 3.60% (95% CI:3.31-3.89), 1.37% (95% CI:1.09-1.66), and 1.37% (95% CI:1.09-1.66), respectively.

### 3.4 APC effects on BC mortality attributable to MRs

**Figure 3:**
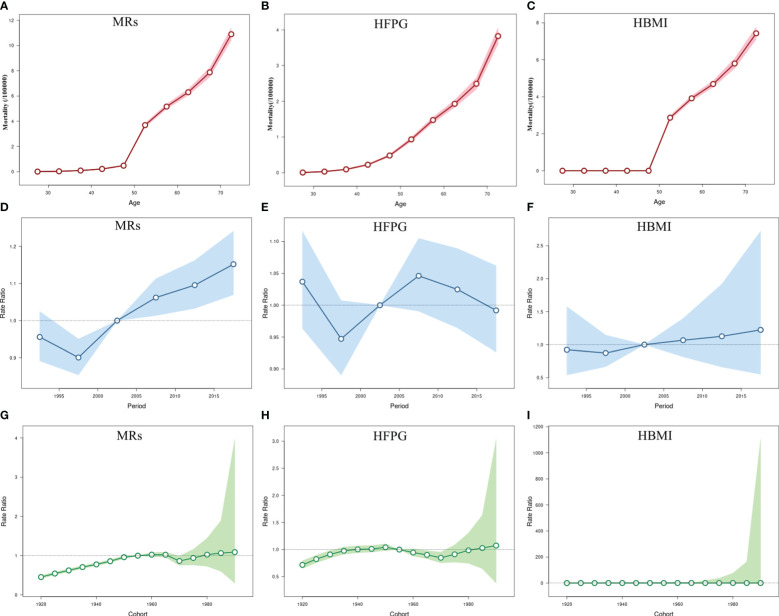
The age-period-cohort (APC) results of BC attributable to MRs (HFPG and HBMI) in China from 1990 to 2019. **(A–C)** Fitted longitudinal age curves of BC mortality (per 100,000) attributable to MRs (HFPG and HBMI); **(D–F)** Rate ratio of each period compared with the reference (2000-2004) adjusted for age and nonlinear cohort effects and the corresponding 95% CI; **(G–I)** Rate ratio of each cohort compared with the reference (cohort 1950-1955) adjusted for age and nonlinear period effects and the corresponding 95% CI. The dots and shaded areas denote mortality or rate ratios and their corresponding 95%CI.

The impact of MRs on BC mortality was most noticeable among individuals aged 50 and above, with the most significant rise observed specifically in the 50-55 age bracket (3.69%; 95% CI: 3.54-3.84 (**Figure 3A**)), similar to the effect attributed to HBMI. Interestingly, mortality due to HFPG positively correlated with age, showing an increase within the same birth cohort (**Figures 3A–C**). The RR value for the time period effect of BC mortality linked to HBMI and HFPG consistently exceeded one from 2005 onwards across all age groups. The stage-specific impact indicated a steady upward trend in mortality attributable to MRs since 2005, indicating a progressively negative impact on overall women’s health with societal development attributable to MRs. In contrast to deaths related to HBMI, the Relative Risk (RR) of the period effect attributed to HFPG remained higher until 2004 and gradually decreased thereafter, suggesting a decline in HFPG-associated risk in the overall women since 2005 (**Figures 3D–F**). Similarly, the cohort effect of BC mortality attributed to MRs gradually increased throughout the study period. Cohorts born after 1920 steadily rose, with a slight decline in the cohort between 1965 and 1970. Individuals born after 1960 have a higher relative risk of death attributable to MRs, particularly in the female population born after 1980, which had a greater than 1 and increasing RR for BC mortality. There was a slight downward trend in RR values for the cohort effect of BC attributable to HBMI among the post-1980 birth cohort (**Figures 3G–I**). All age, period, and cohort effects were statistically significant; detailed results are provided in **Table S2**.

## 4 Discussion

The study comprehensively examined the trends in BC mortality linked to MRs (HFPG and HBMI) among Chinese women aged over 25 from 1990 to 2019, utilizing longitudinal data sourced from GBD 2019. Current evidence has increasingly highlighted the escalating impact of aggregated MRs or metabolic syndrome on female BC mortality, particularly within Asian demographics (30–32). Our findings corroborate this trend, revealing a 4.2-fold surge in MRs-induced deaths among Chinese women from 1990 to 2019, accompanied by an 85% proportional increase in mortality. Moreover, BC-related mortality attributed to both HFPG and HBMI increased, with HBMI demonstrating a more pronounced impact. This increase may be attributed to the accelerated population aging, partly evident in the shift from 50% to 90% in “aging” cities in a study encompassing 287 Chinese cities between 2000 and 2010 (33). Additionally, rapid economic and social developments might have influenced dietary and behavioral changes due to increased income, amplifying metabolic risk factors accumulation.

The risk of BC mortality linked to MRs (HFPG and HBMI) shows a rapid increase among middle-aged and older women, particularly those aged over 50. The evidence supporting increasing age and HBMI and HFPG as potentially high-risk factors for BC underscores the strong correlation between these factors and heightened mortality (34–38). The significant interaction of age and HBMI with BC mortality was also confirmed in a longitudinal study of Chinese women (39). As an abnormal metabolic state, HFPG has been proposed as an independent risk factor for BC along with age (40–43). It could be due to that women over 50 are more prone to menopause, wherein the impact of metabolic abnormalities on BC risk becomes particularly dependent on menopause status (44). Furthermore, HBMI presence has been associated with hormone receptor-positive subtypes in postmenopausal women, including those who have never used menopausal hormone therapy (45–48). A significant dose-response association exists between BMI and postmenopausal BC incidence or death, aligning with the continued increase in overweight obesity rates among Chinese adult women (49–52). Moreover, HBMI was identified as a confounding factor in the correlation between HFPG and BC (53, 54). A meta-analysis of diabetic women and BC risk also showed that studies including BMI had higher RRs in subgroup analyses (55). BC is usually detected later in obese patients (56). Hyperinsulinemia and insulin resistance caused by HBMI are generally predominantly manifested by HFPG (57), an interrelationship that is particularly evident in middle-aged and older women. The established association between BC risk and HFPG, particularly in older postmenopausal age and those with HBMI, further underlines the importance of the HBMI and HFPG interactions. Secondly, breast density is inversely associated with age and HBMI, whereas HBMI and age significantly increase the risk of abnormal FPG (OR=1.44) (58, 59). A decrease in breast tissue density implies an increase in breast adipose tissue, which would result in less resistance encountered by X-rays when penetrating the breast tissue, leading to a rise in the sensitivity of the X-rays. On the other hand, the increased depth of X-ray penetration into breast tissue makes it easier to detect subtle structures and abnormalities in the breast, thus improving the accuracy of X-ray diagnosis. The effects of HBMI and HFPG on women’s breast density have somewhat increased BC detection rates and have also had an impact on the risk of metabolically attributable BC deaths in middle-aged and older women (60, 61). Our results implied that MRs remain a significant challenge to Chinese women’s health. Hence, middle-aged and older women constitute high-risk groups and should prioritize the potential adverse impact of the risk of BC death dominated by MRs (HFPG and HBMI), necessitating robust health education initiatives targeted at key populations to mitigate the risk of mortality from associated diseases.

The relative risk of BC mortality associated with MRs was higher and has continued to increase in all age groups since 2005 according to the results of the period effect study. Women’s attitudes have changed dramatically with late marriage and childbearing, advanced childbearing age, and non-breastfeeding, leading to an increasing risk of BC in women with the development of society (62, 63). Lifestyle changes have likewise brought about earlier age of menarche, later age of first pregnancy and menopause, and long-term use of oral contraceptives or hormone therapy (64, 65), which together have driven the constant changes in women’s metabolic levels and have contributed mainly to the increasing mortality rate of BC attributable to MRs. Throughout the study period, the RR value of the period effect caused by HBMI tended to increase yearly, while the product caused by HFPG tended to rise and then decrease. HFPG is usually manifested in the form of diabetes mellitus, and the Chinese government has included diabetes mellitus as a primary chronic disease in the essential public service program since 2009 (66). It has issued several management manuals and prevention and treatment guidelines, which have greatly controlled and improved HFPG levels in the Chinese population. Levels of HFPG in the population also coincide with the time point of decline that occurred in our study. In addition, this difference may be because Chinese women generally prefer to know their health status through blood glucose levels rather than BMI. Although weight reduction is more cost-effective for women’s health, the broad selectivity, acceptability, sustainability, and predictability of the effects of glucose reduction tend to make glucose reduction the preferred choice for most women, which may explain the changes in the RR values of the cycling effect of BC mortality caused by the two abnormal metabolic statuses in this study.

Our study observed that the relative risk of mortality was greater in the birth cohort of women after the 1960s in analyzing the cohort effect of MRs on BC mortality of Chinese women. As we have mentioned, the rapid economic and social development has significantly impacted behavior and dietary patterns, such as sedentary lifestyle, alcohol consumption, circadian rhythm disruption, irrational diet, etc. (67, 68), rendering metabolic factors a significant threat to BC mortality among women, particularly affecting younger birth cohorts. In 2019, MRs ranked highest among all risk factors contributing to BC mortality, with HFPG showing an increased ranking compared to 1990, while HBMI maintained its third-place position in both years (**Figure S2**). It’s interesting that a slight decline was observed in the RR of the BC cohort effect attributed to HBMI in the post-1980 birth cohort. This trend might be associated with heightened health literacy and increased body image concerns among the younger generation, among other potential factors. This discovery could offer a promising avenue for behavioral improvements to reduce BC mortality linked to MRs among young women.

The study has both strengths and limitations. The strengths are that the definition and identification approach of metabolic risk factors associated with BC were adopted from those in GBD, which warranted the data being comparable across GBD-related studies. Moreover, the study population was selected from Chinese women aged 25 years or older, which is more representative of the age group of the study participants. Meanwhile, the effects of age, period, and cohort on the risk of BC mortality attributable to MRs from a historical epidemiological perspective were estimated using the APC model among participants in the study. However, limitations are also worthy of attention. Firstly, the data in our study were derived from the GBD study, which implies that the potential bias in the GBD study was also present in our study (20, 21). Secondly, this study failed to comprehensively analyze the changes in the effects of metabolic factors on BC mortality before and after menopause as BC is closely related to hormone levels. Thirdly, the impact of other metabolic factors and interactions between factors on BC mortality was not considered. In addition, the analysis of GBD data for mortality risk factors needs to be further improved and strengthened in the future (17).

## 5 Conclusions

In summary, an increasing trend in BC mortality attributable to MRs (HFPG and HBMI) has been observed from 1990 to 2019 among Chinese women aged 25 years or older. Participants’ age, period, and birth cohort all contribute to BC mortality attributable to MRs (HFPG and HBMI). This study has important public health implications for preventing and treating BC in the Chinese female population. It identifies priority groups for prevention and treatment based on the findings, thereby offering an evidence-based approach to mitigate BC mortality among Chinese women and achieve the objectives of the Healthy China strategy.

## Data availability statement

Publicly available datasets were analyzed in this study. This data can be found here: http://ghdx.healthdata.org.

## Author contributions

TZ: Data curation, Software, Writing – original draft, Writing – review & editing. SS: Data curation, Writing – review & editing. TX: Formal analysis, Supervision, Writing – review & editing. QH: Data curation, Software, Writing – review & editing. YF: Methodology, Supervision, Writing – review & editing. WW: Formal analysis, Methodology, Writing – review & editing. HuY: Funding acquisition, Methodology, Supervision, Writing – review & editing. XH: Funding acquisition, Data curation, Validation, Writing – review & editing. NZ: Conceptualization, Supervision, Writing – review & editing. HaY: Funding acquisition, Supervision, Writing – review & editing.
