# Supplementary material for: Trends in breast cancer mortality attributable to metabolic risks in Chinese women from 1990 to 2019: an age-period-cohort analysis
Source: Front Oncol. 2024 Apr 16;14:1369027. doi: 10.3389/fonc.2024.1369027 (PMC11058724; doi:10.3389/fonc.2024.1369027)
Supplement: Supplementary file 1 [file DataSheet_1.docx]

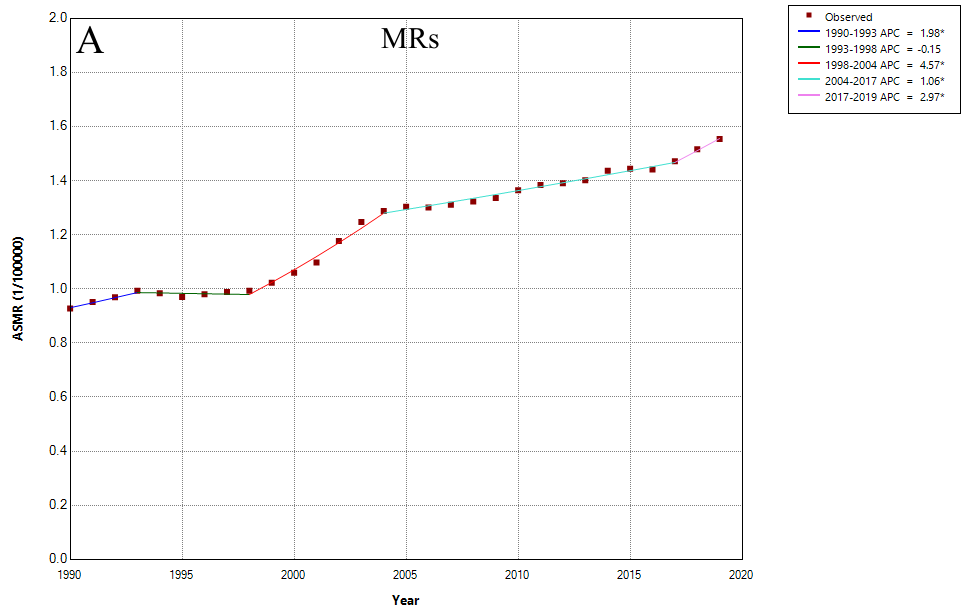

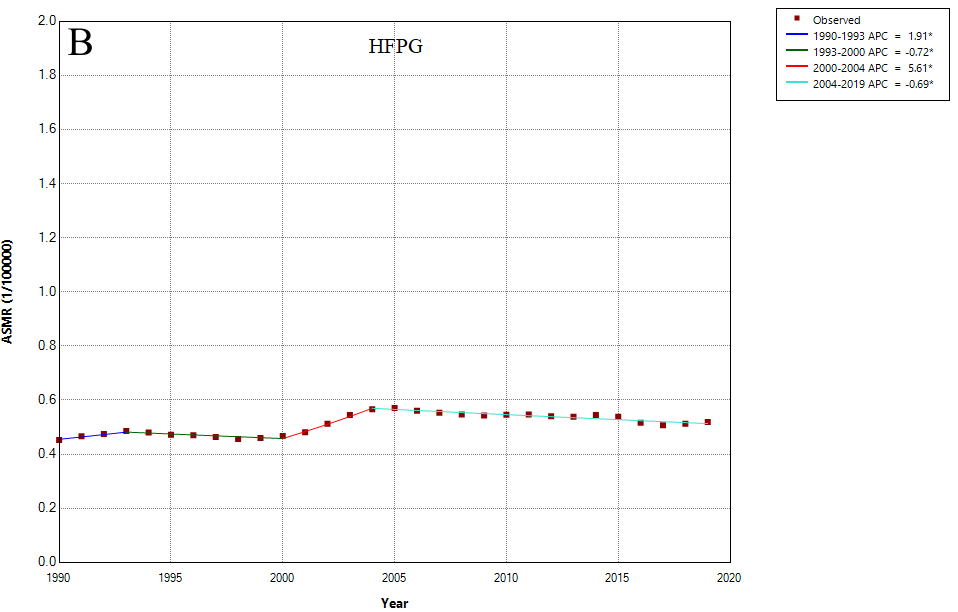

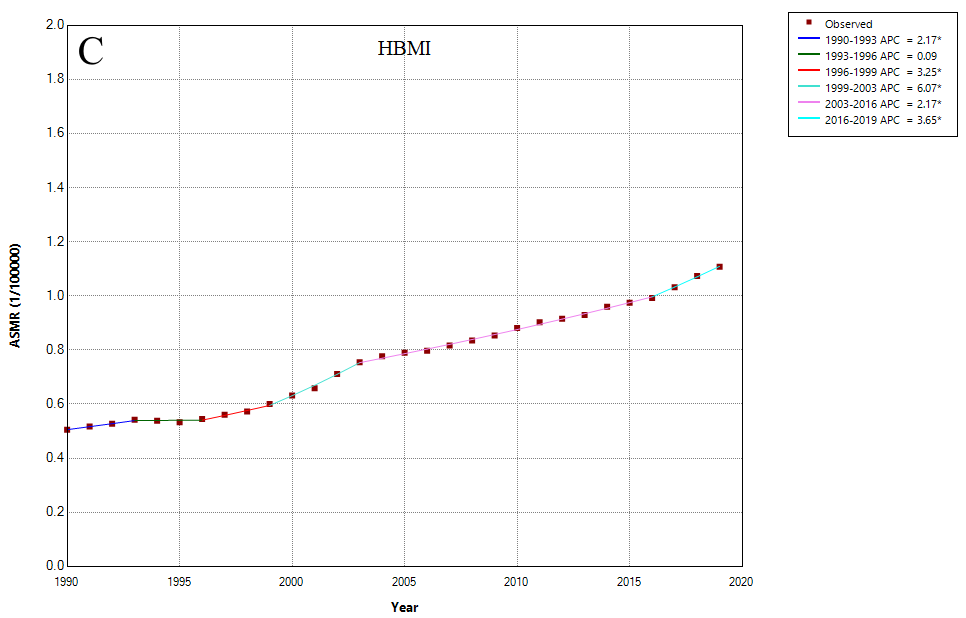


**Figure S1.** Joinpiont regression analysis of trends in the ASMR of BC attributable to MRs (HFPG and HBMI) in China, 1990 to 2019.

*Indicates that Annual Percent Change (APC) is significantly different from zero at the alpha=0.05 level


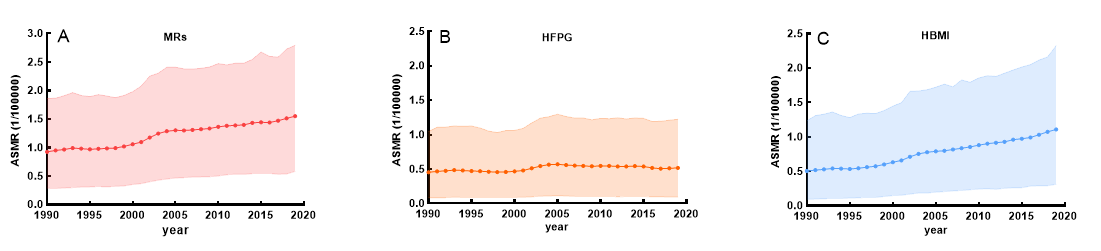


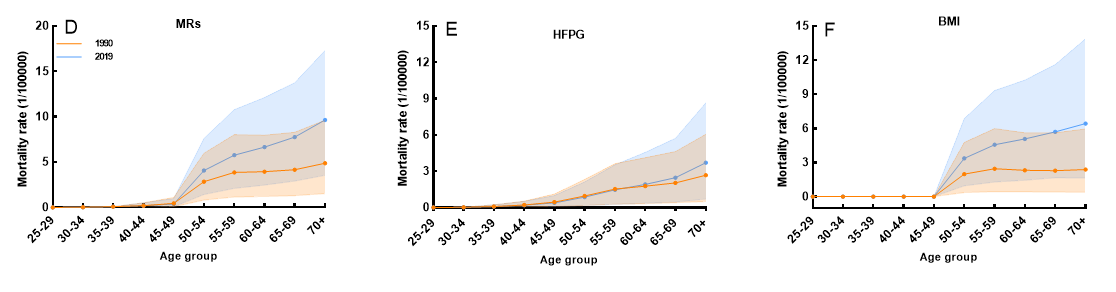


**Figure S2.** Trends in BC mortality attributable to MRs (HFPG and HBMI).

**(A-C)** Annual Trends in ASMR for BC attributable to MRs (HFPG and HBMI), 1990-2019.

**(D-F)** Age-specific mortality rate attributable to MRs (HFPG and HBMI), 1990 vs. 2019.

**Table S1.** The Mortality and annual trends of BC by age group attributable to MRs (HFPG and HBMI), 1990 vs. 2019

| Age group | MRs | | | HFPG | | | HBMI | | |
| --- | --- | --- | --- | --- | --- | --- | --- | --- | --- |
|  | 1990 | 2019 | AAPC% (95%CI) | 1990 | 2019 | AAPC% (95%CI) | 1990 | 2019 | AAPC% (95%CI) |
| 25~29 | 0.005(0.001,0.01) | 0.01(0.001,0.01) | 0.99*(0.31~1.57) | 0.005(0.001,0.01) | 0.01(0.001,0.01) | 0.99*(0.31~1.57) | 0(0,0) | 0(0,0) |  |
| 30~34 | 0.02(0.004,0.06) | 0.03(0.004,0.07) | 0.51*(0.07~0.97) | 0.02(0.004,0.06) | 0.03(0.005,0.07) | 0.51*(0.07~0.98) | 0(0,0) | 0(0,0) |  |
| 35~39 | 0.09(0.02,0.22) | 0.09(0.01,0.23) | -0.01(-0.10~0.09) | 0.09(0.02,0.22) | 0.09(0.01,0.23) | -0.01(-0.10~0.09) | 0(0,0) | 0(0,0) |  |
| 40~44 | 0.21(0.04,0.53) | 0.20(0.03,0.52) | -0.10*(-0.17~ ~0.03) | 0.21(0.04,0.53) | 0.2(0.04,0.52) | -0.10*(-0.17~ ~0.03) | 0(0,0) | 0(0,0) |  |
| 45~49 | 0.45(0.08,1.12) | 0.40(0.07,0.99) | -0.57*(-0.81~ ~0.23) | 0.45(0.08,1.12) | 0.40(0.07,0.99) | -0.57*(-0.81~ ~0.22) | 0(0,0) | 0(0,0) |  |
| 50~54 | 2.85(0.81,5.98) | 4.09(1.44,7.62) | 1.20*(1.15~1.25) | 0.95(0.17,2.33) | 0.87(0.16,2.12) | -0.32*(-0.44~ ~0.23) | 1.98(0.36,4.78) | 3.37(0.96,6.87) | 1.86*(1.81~1.91) |
| 55~59 | 3.87(1.16,8.03) | 5.77(2.11,10.79) | 1.35*(1.24~1.50) | 1.54(0.28,3.63) | 1.46(0.27,3.54) | -0.26*(-0.40~ ~0.77) | 2.46(0.44,5.99) | 4.57(1.29,9.34) | 2.14*(2.03~2.27) |
| 60~64 | 3.94(1.23,7.97) | 6.66(2.47,12.14) | 1.74*(1.62~1.84) | 1.77(0.33,4.12) | 1.92(0.35,4.57) | 0.25*(0.12~0.37) | 2.32(0.43,5.62) | 5.07(1.45,10.26) | 2.69*(2.56~2.79) |
| 65~69 | 4.17(1.30,8.3) | 7.77(2.91,13.77) | 2.09*(1.96~2.22) | 2.04(0.4,4.63) | 2.48(0.45,5.73) | 0.64*(0.49~0.80) | 2.29(0.41,5.63) | 5.71(1.67,11.63) | 3.15*(3.04~3.27) |
| 70+ | 4.89(1.53,9.62) | 9.65(3.54,17.32) | 2.31*(2.25~2.38) | 2.68(0.5,6.06) | 3.72(0.70,8.65) | 1.06*(0.96~1.17) | 2.39(0.4,5.96) | 6.43(1.65,13.89) | 3.46*(3.40~3.51) |

*Indicates that Average Annual Percentage Change (AAPC) is significantly different from zero at the alpha=0.05 level

**Table S2**. The wald χ2 test of age, period and cohort effects

|  | MRs | | | HFPG | | | HBMI | | |
| --- | --- | --- | --- | --- | --- | --- | --- | --- | --- |
|  | χ2 | df | P~value | χ2 | df | P~value | χ2 | df | P~value |
| NetDrift = 0 | 14.68 | 1 | 0.0001 | 0.03 | 1 | 0.8656 | 0.22 | 1 | 0.6373 |
| All Age Deviations = 0 | 3675.67 | 8 | ＜0.001 | 621.57 | 8 | ＜0.001 | 142.21 | 8 | ＜0.001 |
| All Period Deviations = 0 | 15.45 | 4 | 0.0038 | 15.52 | 4 | 0.0037 | 17.78 | 4 | 0.0014 |
| All Cohort Deviations = 0 | 106.77 | 13 | ＜0.001 | 81.60 | 13 | ＜0.001 | 96.43 | 13 | ＜0.001 |
| All Period RR = 1 | 32.15 | 5 | ＜0.001 | 15.52 | 5 | 0.0084 | 18.02 | 5 | 0.0029 |
| All Cohort RR = 1 | 770.25 | 14 | ＜0.001 | 118.34 | 14 | ＜0.001 | 1489.29 | 14 | ＜0.001 |
| All Local Drifts = Net Drift | 103.65 | 10 | ＜0.001 | 80.11 | 10 | ＜0.001 | 91.48 | 10 | ＜0.001 |


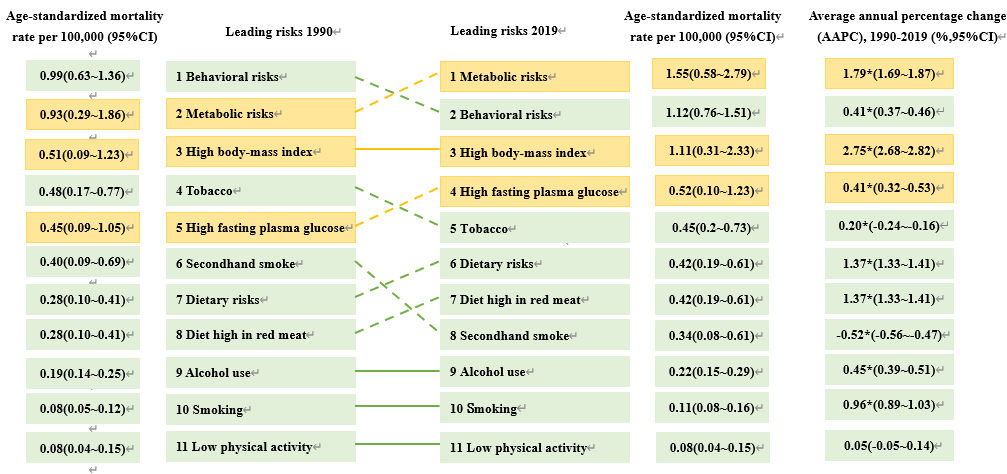


**Figure S3.** The rank of all risk factors for BC in Chian in 1990 and 2019.
